# Spontaneous Thrombosis of a Giant Cavernous Internal Carotid Artery Aneurysm and Parent Vessel Occlusion in a Patient With Bilateral Cavernous Internal Carotid Artery Aneurysms

**DOI:** 10.7759/cureus.35231

**Published:** 2023-02-20

**Authors:** Mira Salih, Michael Young, Max Shutran, Philipp Taussky, Christopher S Ogilvy

**Affiliations:** 1 Neurosurgery, Beth Israel Deaconess Medical Center, Harvard Medical School, Boston, USA

**Keywords:** flow diversion, spontaneous aneurysm occlusion, giant thrombosed aneurysm, carotid artery occlusion, cavernous carotid artery aneurysm

## Abstract

Spontaneous thrombosis of giant aneurysms is a well-reported phenomenon. However, reports of complete occlusion of the aneurysm and parent vessel are scarce. Here, we describe the case of a patient with spontaneous thrombosis of a giant cavernous internal carotid artery (ICA) aneurysm and occlusion of the ICA. A 59-year-old female initially presented with frequent headaches and was otherwise completely neurologically intact. Magnetic resonance angiography (MRA) demonstrated a giant, partially thrombosed right cavernous ICA aneurysm. She was also found to have a contralateral left-sided intracavernous aneurysm. Cerebral angiogram revealed a giant, partially thrombosed right cavernous segment ICA aneurysm measuring 27.1 x 32.4 mm with slow, turbulent flow within the lesion. The patient was started on aspirin 325 mg and a dexamethasone taper with plans for follow-up flow diversion for treatment of the right cavernous ICA aneurysm. The patient presented three months later with worsening headaches, and on examination was found to have anisocoria (right > left) with a nonreactive right pupil as well as cranial nerve III/IV palsies, and facial edema. There was no evidence of intracranial hemorrhage or ischemia seen on head computed tomography (HCT). The diagnostic cerebral angiogram demonstrated complete occlusion of the right ICA at the carotid bifurcation with no filling of the giant right cavernous ICA aneurysm and a stable left cavernous ICA aneurysm. Although the exact mechanism of simultaneous thrombosis of the aneurysm and its parent artery remains unclear, it is likely due to stagnant flow. The presence of cranial nerve palsies was most likely secondary to acute edema of the lesion after thrombus formation. There was no evidence of ischemic symptoms due to collateral flow across a patent anterior communicating artery.

## Introduction

Most cavernous internal carotid artery (ICA) aneurysms have been reported to have a benign natural history and low cumulative rupture rate [1]. However, large cavernous aneurysms may rapidly grow due to the aneurysm size being a significant risk for aneurysmal growth. Because of the anatomic location of the cavernous ICA within the cavernous sinus, these aneurysms can cause cavernous sinus syndrome. Cavernous sinus syndrome often manifests as ophthalmoplegia, visual changes, ptosis, proptosis, facial pain or numbness, and Horner’s syndrome [2,3]. Spontaneous thrombosis of large aneurysms is a well-known phenomenon, with the frequency reported to be 13%-60% [4-8]. However, reports of complete occlusion of the aneurysm and parent vessel are scarce. Herein, we present a case of spontaneous thrombosis of a giant cavernous aneurysm and angiographically documented occlusion of the internal carotid artery.

## Case presentation

**Figure 1:**
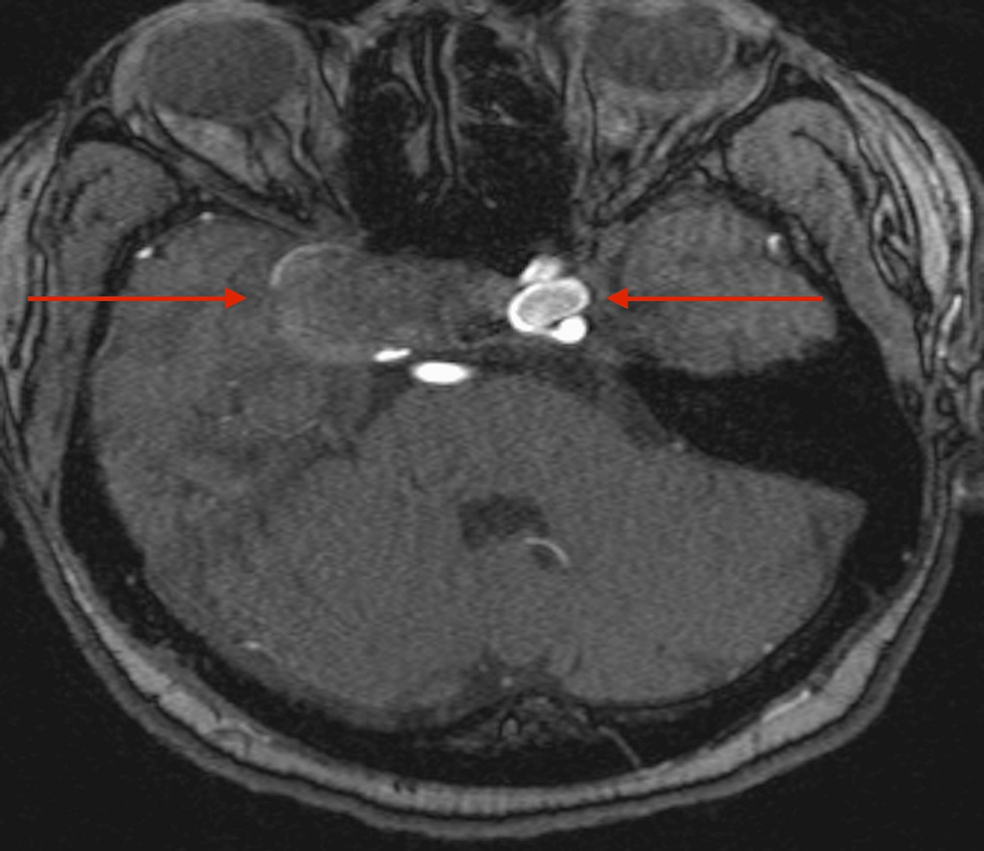
MRA showing a giant, partially thrombosed, right cavernous ICA aneurysm and a large, left cavernous ICA aneurysm MRA, magnetic resonance angiography; ICA, internal carotid artery

A 59-year-old female with a history of hypertension and hyperlipidemia initially presented with frequent headaches and was neurologically intact on examination. Magnetic resonance angiography (MRA) demonstrated a giant, partially thrombosed right cavernous ICA aneurysm measuring approximately 2.7 cm and a contralateral, left-sided cavernous ICA aneurysm measuring approximately 1.3 cm (Figure 1).

**Figure 2:**
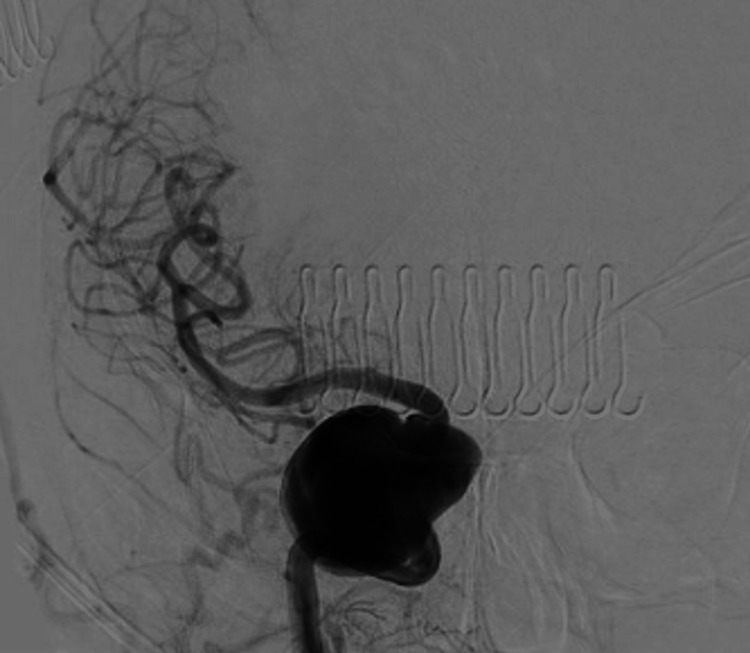
Anterior posterior cerebral angiogram demonstrating a giant, right cavernous ICA aneurysm ICA, internal carotid artery

**Figure 3:**
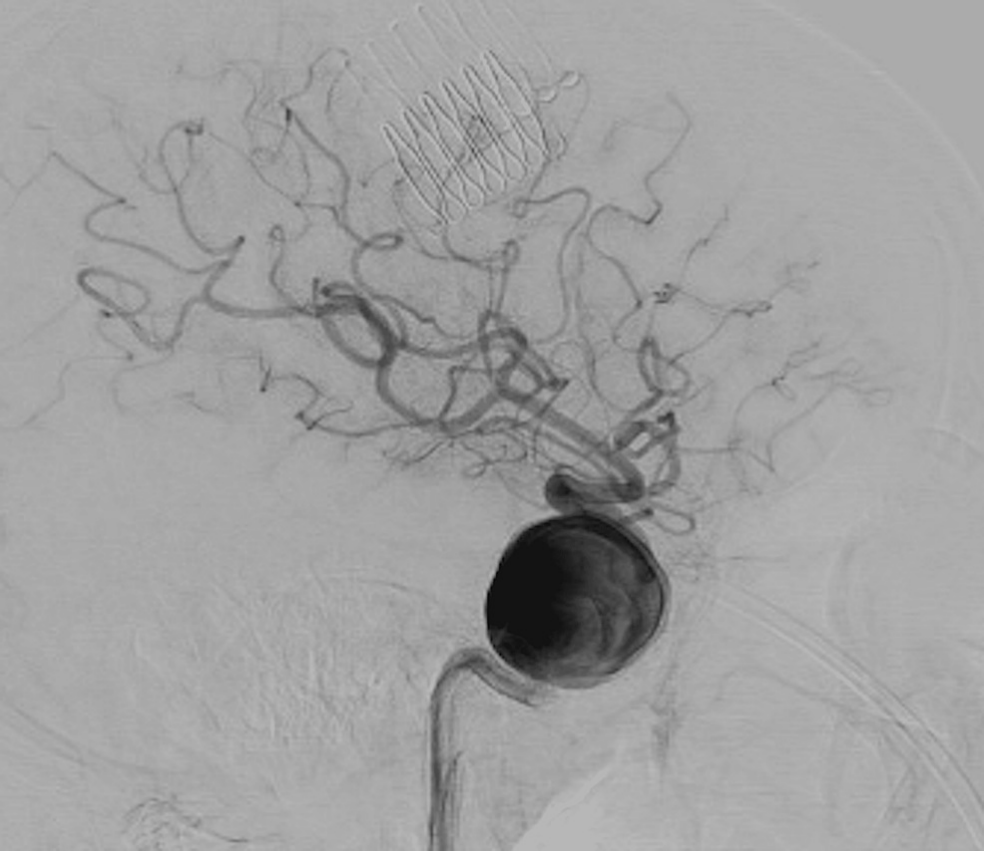
Lateral cerebral angiogram demonstrating a giant, right cavernous ICA aneurysm ICA, internal carotid artery

**Figure 4:**
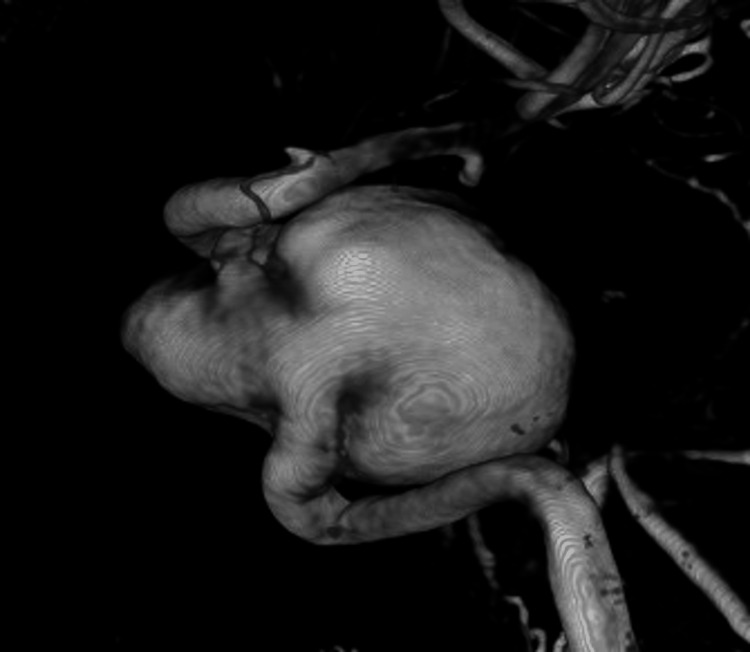
3D reconstruction demonstrating a giant, right cavernous ICA aneurysm ICA, internal carotid artery

A cerebral angiogram revealed a giant, partially thrombosed, right cavernous ICA aneurysm measuring 27.1 x 32.4 mm with slow, turbulent flow within the lesion (Figures 2-4).

**Figure 5:**
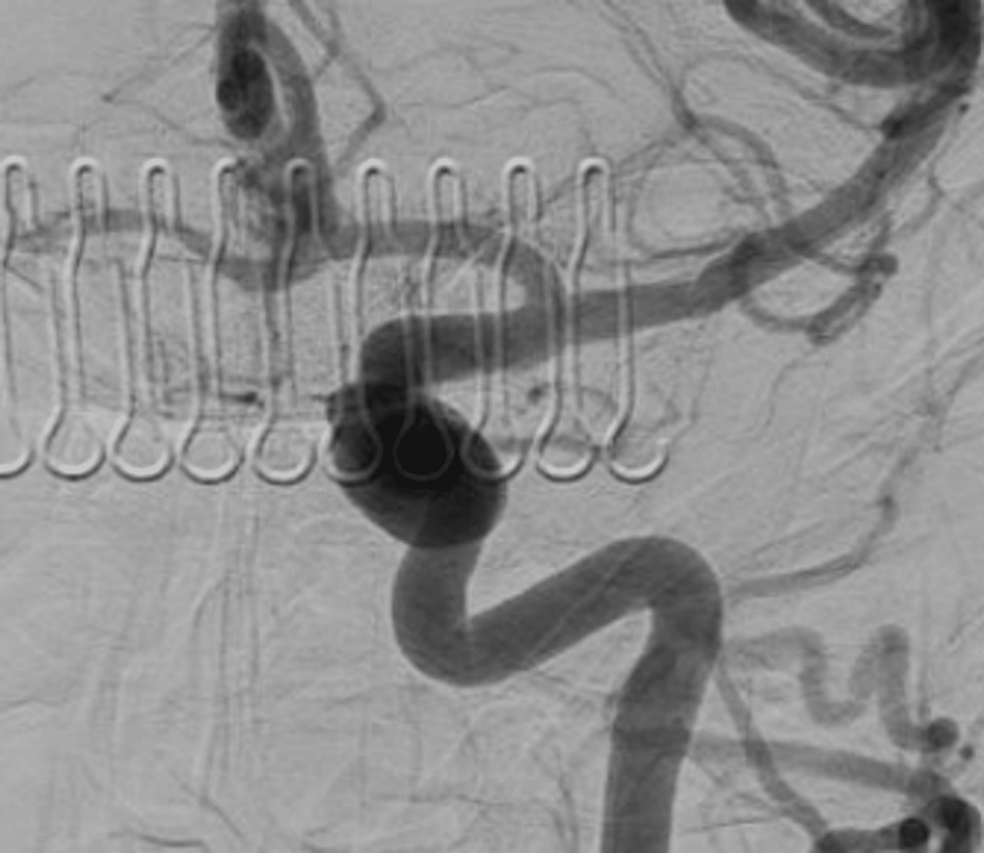
Anterior posterior cerebral angiogram demonstrating a large, left cavernous ICA aneurysm ICA, internal carotid artery

**Figure 6:**
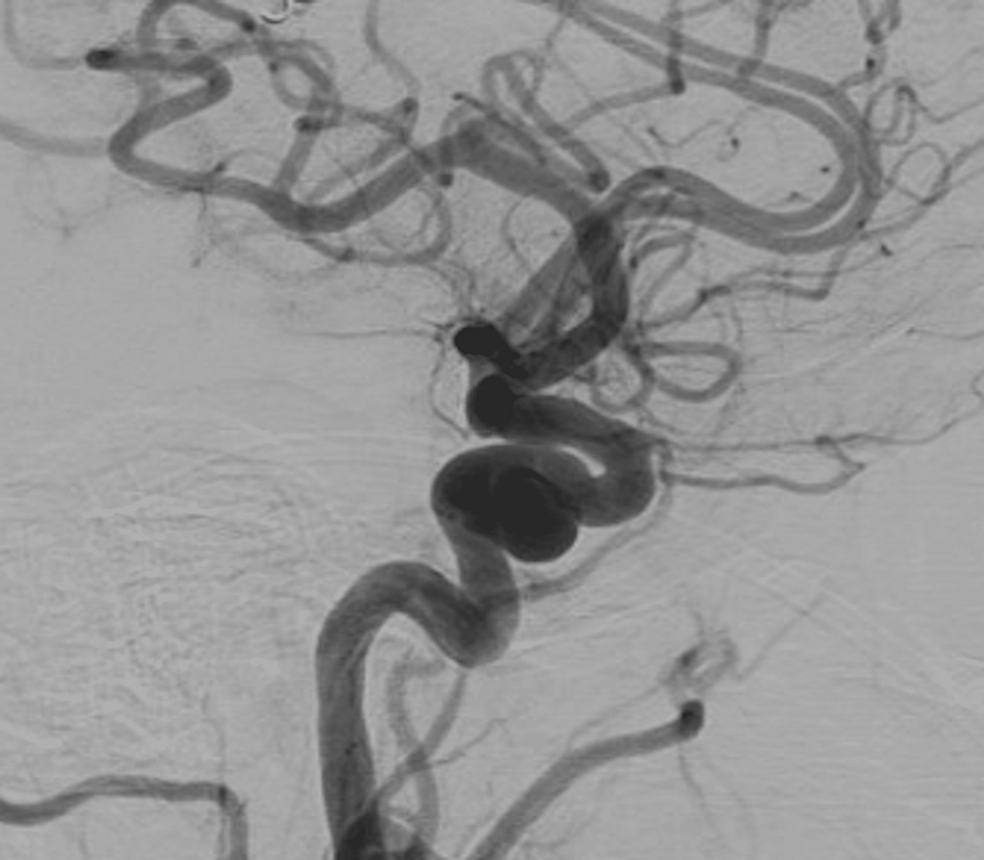
Lateral cerebral angiogram demonstrating a large, left cavernous ICA aneurysm ICA, internal carotid artery

**Figure 7:**
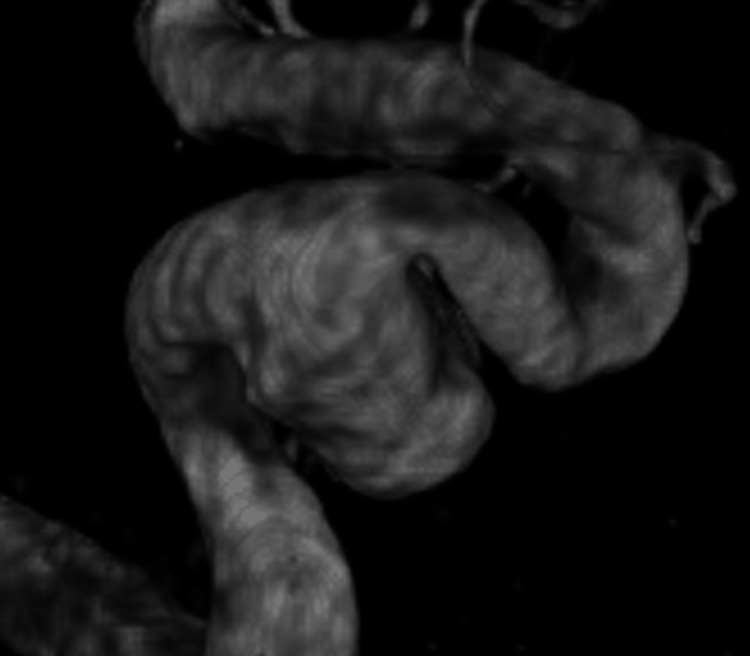
3D reconstruction demonstrating a large, left cavernous ICA aneurysm ICA, internal carotid artery

The right anterior cerebral artery was atretic and the right distal anterior cerebral artery filled from the dominant left A1 segment of the anterior cerebral artery. On the diagnostic cerebral angiogram, the left cavernous ICA aneurysm measured 12.5 x 7.3 mm (Figures 5-7).

The patient was discharged home on aspirin 325 mg and a dexamethasone taper and with plans for flow diversion of the giant, right cavernous ICA aneurysm in the short-term follow-up.

**Figure 8:**
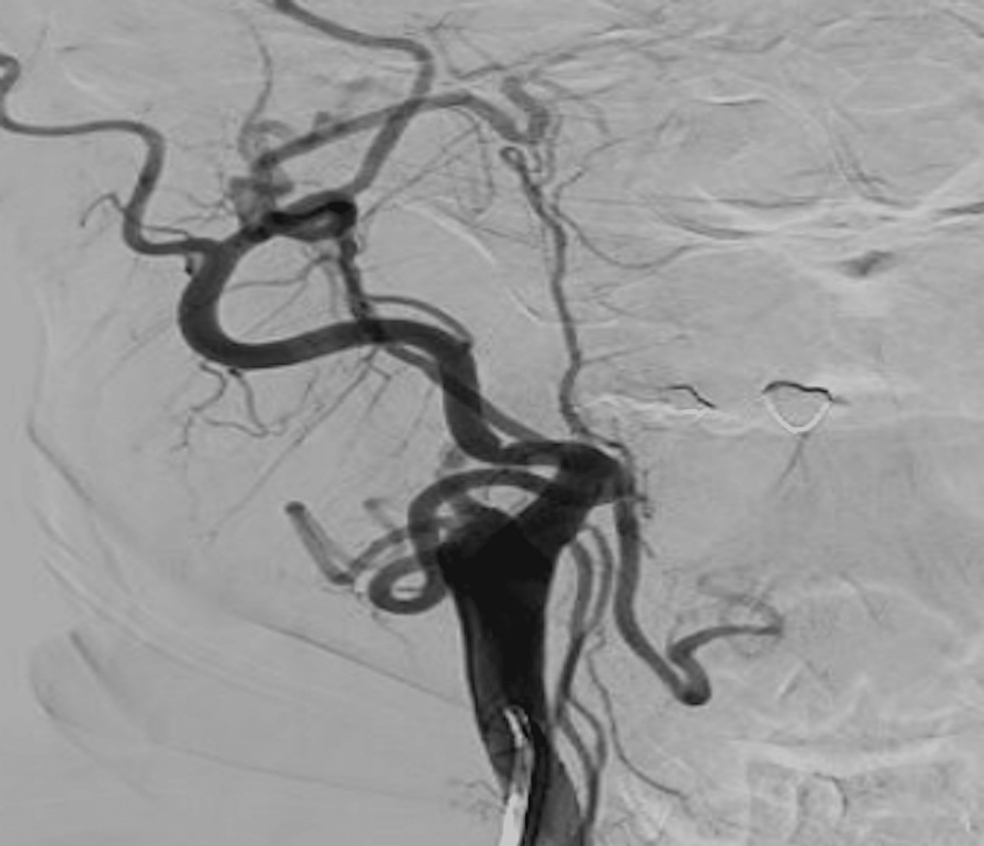
Anterior posterior cerebral angiogram demonstrating spontaneous complete occlusion of the right ICA and giant, right cavernous ICA aneurysm ICA, internal carotid artery

**Figure 9:**
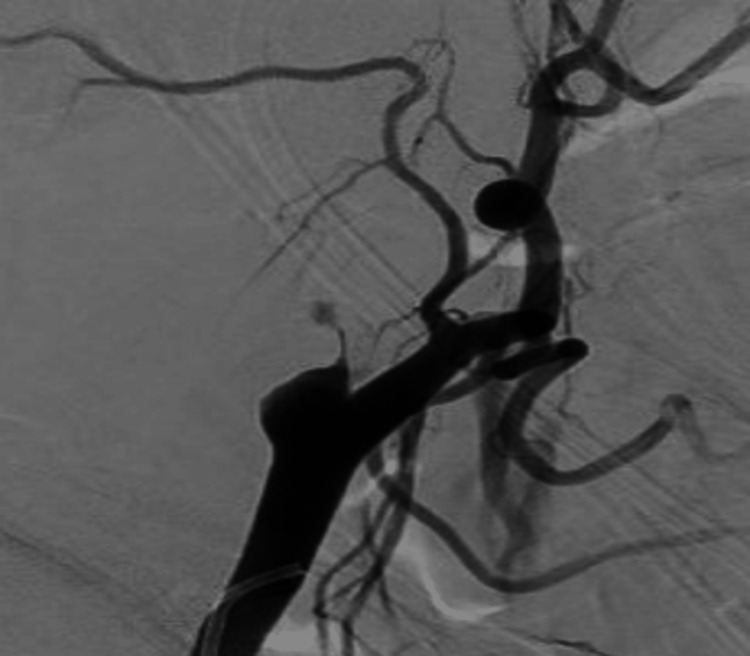
Lateral cerebral angiogram demonstrating spontaneous complete occlusion of the right ICA and giant, right cavernous ICA aneurysm ICA, internal carotid artery

Prior to the flow diversion treatment, she presented with worsening headaches, and on examination was found to have anisocoria (right > left) with a nonreactive right pupil, as well as cranial nerve III/IV palsies and facial edema. No acute findings were seen on head CT. The cerebral angiogram demonstrated complete occlusion of the right ICA at the carotid bifurcation with no filling of the giant, right cavernous ICA aneurysm (Figures 8, 9).

**Figure 10:**
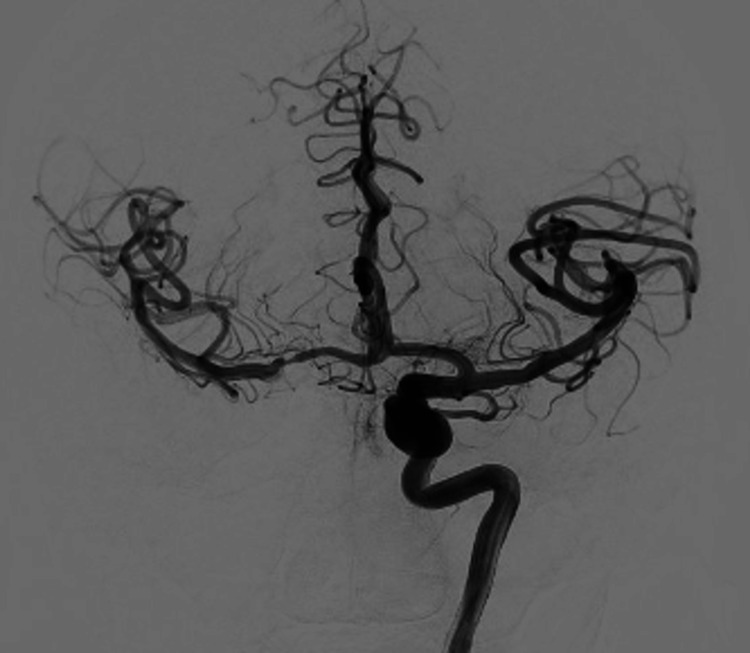
Anterior posterior cerebral angiogram demonstrating filling of right anterior circulation via collateral flow from the left ICA across the anterior communicating artery ICA, internal carotid artery

The right anterior circulation filled from a patent left internal carotid artery across the anterior communicating artery as well as right external carotid artery collateral filling retrograde across the right ophthalmic artery (Figure 10).

**Figure 11:**
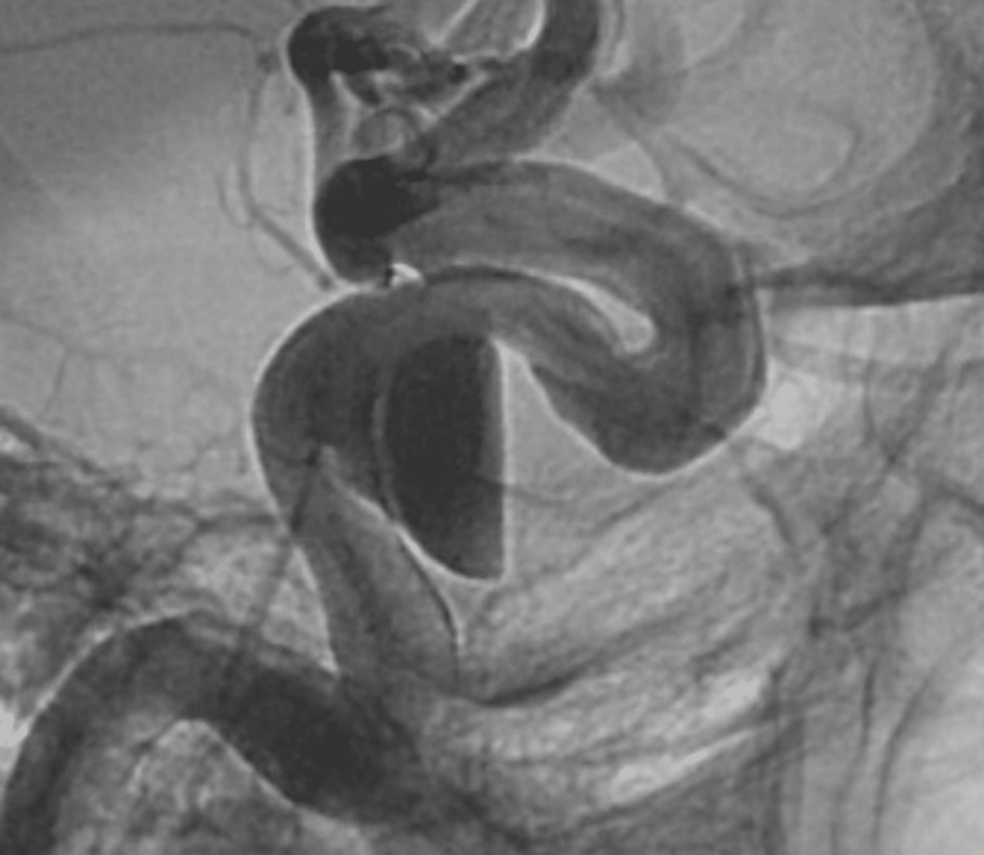
Lateral unsubtracted cerebral angiogram post-pipeline embolization of the large, left cavernous ICA aneurysm ICA, internal carotid artery

Due to concerns of spontaneous thrombosis of the contralateral ICA and therefore the entire anterior circulation, the decision was made to treat the left cavernous ICA aneurysm. The patient was transitioned to aspirin 81 mg daily and started on Brilinta 90 mg twice a day. A 5.00 x 20 mm pipeline embolization device (Medtronic, Dublin, Ireland) was successfully deployed across the aneurysm without any intra-operative complications (Figure 11).

The patient was continued on dual antiplatelet therapy with aspirin 81 mg daily and ticagrelor 90 mg twice a day with a plan for a repeat diagnostic cerebral angiogram one year after the procedure.

## Discussion

Spontaneous partial thrombosis of giant intracranial aneurysms is a well-reported phenomenon with a relatively high incidence rate [8,9]. Complete occlusions have been reported for middle cerebral artery, posterior cerebral artery and internal carotid aneurysms [10-12]. However, reports of complete occlusion of the cavernous ICA aneurysm and parent vessel are scarce. Although the exact mechanism of simultaneous thrombosis of the aneurysm and its parent artery remains unclear, it is reported that it generally takes place in large aneurysms. Some studies postulate that stress on the aneurysmal wall with subsequent endothelial damage is the main cause of aneurysmal thrombosis. Others argue that changes in hemodynamics due to stagnant or turbulent flow cause the formation of thrombus in an aneurysm, which is likely the case in our patient [8,9]. Hypercoagulable state, long-standing aneurysm without treatment and dome to neck ratio have been thought to be contributing factors as well for thrombosis of aneurysms [8].

Isolated complete thrombosis of the aneurysm may be a welcome phenomenon; however, simultaneous thrombosis of the parent artery can cause undesirable neurologic complications. Therefore, it is important to understand the clinical manifestation of spontaneous giant aneurysm thrombosis. In our case, cranial nerve palsies were most likely secondary to acute edema of the lesion after thrombus formation and no other deficits were incurred due to collateral flow from the posterior circulation. Acute parent vessel thrombosis or compression by a thrombosed aneurysm is known to produce ischemic neurological deficits. Thus, we assume the presence of clinical symptoms depends on the rapidity of the parent vessel thrombosis as well as the presence of an adequate cerebrovascular reserve or sufficient collateral circulation.

The cavernous sinus typically is a location for aneurysms where hemorrhage is less common. The patient initially presented in neurologically intact condition; therefore the decision for short-term follow-up treatment was made. However, given the large size of the right-sided lesion in our case, it might erode the sphenoid sinus medially and it would be concerning if hemorrhage did occur. Before making any decision on management strategy in aneurysmal thrombosis, it is important to review the natural history of it. Studies have shown that it is possible to see further aneurysmal growth, distal thromboembolism, persistent mass effect as well as future re-canalization in completely thrombosed intracranial aneurysms as long as the parent arterial flow is maintained. The recanalization rate in spontaneously thrombosed aneurysms is thought to be not as high as it is in coiled aneurysms. In a case report by Perrini et al., a patient was continuously observed without intervention for a thrombosed giant intracavernous aneurysm with spontaneous ipsilateral carotid artery occlusion. The patient initially presented with headache, double vision and trigeminal numbness that resolved after six months [13]. Yamagami et al. treated a partially thrombosed cavernous ICA aneurysm with embolization through flow diversion [14]. In another case study, Ono et al. performed coil embolization of the primitive trigeminal artery and ligation of the ICA at the cervical portion followed by high‑flow bypass from the cervical external carotid artery to the middle cerebral artery in a patient with a large, partially thrombosed cavernous carotid aneurysm that caused repeated embolic stroke in spite of antiplatelet therapy [15]. In a case series of 28 thrombosed giant intracranial aneurysms managed conservatively, Fujita et al. showed that the giant cavernous ICA aneurysms and completely thrombosed aneurysms did not rupture. The authors advised individualized treatment strategy for these aneurysms, while advocating surgery for the rest of the giant intracranial aneurysms [16].

## Conclusions

Here, we have reported a case of spontaneously thrombosed, giant cavernous artery aneurysm with rapid and complete occlusion of the internal carotid artery in a patient with bilateral ICA cavernous segment aneurysms. Although the exact mechanism of simultaneous thrombosis of the aneurysm and its parent artery remains unclear, it is likely due to stagnant flow within the aneurysm. Cranial nerve palsies are most likely secondary to acute edema of the lesion after thrombus formation. Fortunately, no ischemic symptoms developed due to collateral flow from the contralateral internal carotid artery across the anterior communicating artery.
